# Collagen cross-linking: insights on the evolution of metazoan extracellular matrix

**DOI:** 10.1038/srep37374

**Published:** 2016-11-23

**Authors:** Fernando Rodriguez-Pascual, David Anthony Slatter

**Affiliations:** 1 Centro de Biología Molecular “Severo Ochoa” Consejo Superior de Investigaciones Científicas (C.S.I.C.)/Universidad Autónoma de Madrid (Madrid), Madrid, Spain; 2 School of Medicine, University of Cardiff, Cardiff, United Kingdom

## Abstract

Collagens constitute a large family of extracellular matrix (ECM) proteins that play a fundamental role in supporting the structure of various tissues in multicellular animals. The mechanical strength of fibrillar collagens is highly dependent on the formation of covalent cross-links between individual fibrils, a process initiated by the enzymatic action of members of the lysyl oxidase (LOX) family. Fibrillar collagens are present in a wide variety of animals, therefore often being associated with metazoan evolution, where the emergence of an ancestral collagen chain has been proposed to lead to the formation of different clades. While LOX-generated collagen cross-linking metabolites have been detected in different metazoan families, there is limited information about when and how collagen acquired this particular modification. By analyzing telopeptide and helical sequences, we identified highly conserved, potential cross-linking sites throughout the metazoan tree of life. Based on this analysis, we propose that they have importantly contributed to the formation and further expansion of fibrillar collagens.

Collagens constitute a large family of extracellular matrix (ECM) proteins that play a fundamental role in providing the structural integrity and biomechanical properties of different tissues^1^. In vertebrates, 28 types of collagens have been described (I-XXVIII), which are divided into several families, the most important being fibrillar collagens (I-III, V, XI, XXIV, and XXVII) and basement membrane-forming collagen IV^2^.

Fibrillar collagens form homotrimeric (three identical α-chains) or heterotrimeric (two or three distinct polypeptide chains) molecules. Each α-chain consists of a major uninterrupted triple helical or collagenous domain (characterized by a repetition of G-X-X′ triplets, where G is glycine, X is commonly proline and X′ is commonly hydroxyproline) flanked by N- and C-terminal non-collagenous domains, the N- and C-propeptides.

The biosynthesis of collagen is a highly complicated process involving numerous steps, including chain association and folding, secretion, procollagen processing and cross-linking (see Supplementary Figure 1 for a graphical representation)^3^,^4^. As exemplified for human type I collagen, a heterotrimeric molecule composed of two α1 and one α2 chains, after synthesis on the ribosome and their import into the rough endoplasmic reticulum, collagen chains are subjected to a series of post-translational modifications resulting in the assembly of procollagen chains. These include hydroxylation of specific proline residues, catalyzed by prolyl-4- and prolyl-3-hydroxylases (P4H and P3H enzymes); hydroxylation of specific lysine residues by lysyl hydroxylases (encoded in human by PLOD1-3 genes); N- and O-linked glycosylation, disulphide bonding and prolyl cis-trans isomerization^5^. Association of the three α-chains occurs through a process governed by the C-terminus, and the formation of the triple helix is propagated towards the N-terminal end in a zipper-like fashion to form the procollagen molecule^6^. This precursor molecule is then transported to the Golgi network where it is packaged into specialized secretory vesicles prior to export into the extracellular medium. Formation of fibrils from procollagen chains requires their proteolytic processing. The N- and C-propeptides are cleaved off by metalloproteinases belonging to the ADAMTS (a disintegrin and metalloproteinase with thrombospondin motifs) and BMP1 (bone morphogenetic protein 1)/Tolloid-like families, respectively, yielding the tropocollagen molecule, which retains a short portion of the propeptides termed telopeptides. Then, lysine or hydroxylysine residues within these non-collagenous domains are oxidatively deaminated by LOX, yielding the corresponding aldehydes which constitute the initiation products for the cross-linking formation (see Supplementary Figure 2 for details). Within hours of helix formation, these telopeptide aldehydes spontaneously react with helical lysines or hydroxylysines to form immature cross-links, which further react among them and with remaining lysine or hydroxylysine residues over months/years to form permanent cross-links^5^,^7^,^8^,^9^. The formation of these permanent or mature cross-links is fundamental as they determine the topology of adjacent molecules and contribute to the stiffness of the collagen fibril, where variations in the usage of lysine or hydroxylysine in both telopeptide and helix sites modulate the mechanical properties of the collagen matrix. In fact, defects in PLOD2, the lysyl hydroxylase isoform that specifically acts on lysine residues in collagen telopeptides, are responsible for Brück syndrome, a heritable disorder in the osteogenesis imperfecta spectrum, characterized by severe reduction or elimination of the telopeptide hydroxylysine-derived cross-links, resulting in bone fragility^10^. In addition to lysyl hydroxylases, collagen-associated proteins such as the small leucine-rich protein (SLRP), fibromodulin, have been shown to influence collagen cross-linking, as recently evidenced in fibromodulin-deficient mice^11^,^12^.

Fibrillar collagens are present from sponges to humans, therefore often being associated with metazoan multicellularity and evolution^13^. Given the modular nature of the collagen molecule, different approaches have been used to study its evolutionary history, including phylogenetic or genomic analyses of the propeptides, the triple helix, or even of the intron-exon organization^14^,^15^,^16^,^17^. Regardess of the approach, all authors agree on the appearance of an ancestral fibrillar collagen in the lineage leading up to the Metazoa, given the fact that the choanoflagellate *Monosiga brevicollis*, the sister group to Metazoa, encode proteins with triple helical and C-propeptide (COLFI) sequences in different polypeptides, while collagens present in sponges include both in the same protein chain^14^,^18^. From this point on, two general hypothesis have been raised to explain the evolutionary origin of the three clades (A, B and C) identified in mammalian collagens. On one hand, the ancestor chain might have diverged into two clades (A and B/C) before the poriferan radiation, followed by a further division of B/C clade before the Parazoa/Eumetazoa split. In the second hypothesis, B and C clades are already present in demosponges and their emergence predated metazoan cladogenesis^14^,^19^. Whatever the case, a remarkable expansion of collagen chains occurred in the ancestors of chordates, where gene duplications in the three clades led to the formation of specialized cartilage and bone collagens, a fundamental feature of vertebrate skeletons^20^.

In a recent work, we have proposed that early collagen evolution has been governed by structural requirements intended to preserve the position of spatially sensitive cross-linking sites^21^. On the other hand, we also described the presence of LOX domains, not only in animals, but also in many other eukaryotes, as well as in bacteria and archaea, organisms that are devoid of recognizable fibrillar collagen-based ECM structure^22^. These observations indicate that the origin of LOX enzymes pre-dates the appearance of fibrillar collagens, and suggest that, by co-option of this enzymatic activity, ancestral collagen domains might have evolved into modern fibrillar collagens within the metazoan kingdom. To test this hypothesis, we have surveyed a variety of collagen sequences of different metazoan lineages in order to infer the evolutionary transitions leading to cross-linking sites. By analyzing telopeptide and helical sequences, we identified highly conserved, potential cross-linking sites throughout the metazoan tree of life. Based on this analysis, we propose that they have importantly contributed to the formation and further expansion of fibrillar collagens.

## Results

**Figure 1:**
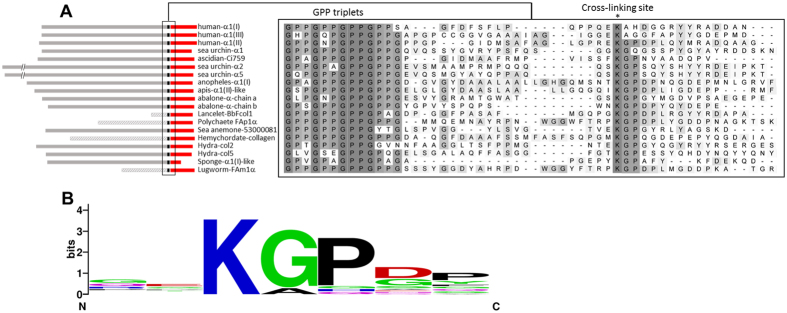
Multiple sequence alignment of potential C-telopeptide cross-linking sites among several metazoan collagens. (**A**) A group of 19 clade A (or A-like) collagens representing different metazoan lineages were aligned using the ClustalW algorithm and potential cross-linking sites within the C-telopeptide sequences were manually checked in the alignment^47^. Left panel shows the location of the aligned segments within the analyzed chains, with the fibrillar collagen C-terminal domain (COLFI, pfam01410) marked in red. Right part shows the alignment, which displays the degree of identity on a gray scale. The position of the GPP triplets, the known motif marking the C-terminal end of the helix region, and the potential cross-linking sites are indicated above the alignment. **(B)** Sequence logo of the position weight matrix of the putative cross-linking sites analyzed in the C-telopeptides collagen chains obtained using WebLogo^48^.

**Figure 2:**
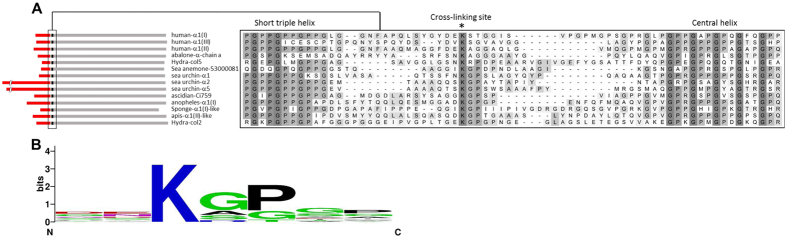
Sequence alignment of N-telopeptide cross-linking sites. (**A**) Metazoan collagen chains used in Fig. 3 were aligned at the N-telopeptide sequences to search for potentially conserved cross-linking sites. Left panel shows the location of the aligned segments, with the N-propeptides marked in red. Right part shows the alignment, which is delimited by the position of the short triple helix and the beginning of the central helical segment. Sequences omitted from the C-propeptide analysis were either incomplete (lancelet, polychaete, lugworm, hemychordate) or lack of significant homology (abalone-α-chain b). (**B**) Sequence logo of the position weight matrix of the putative cross-linking sites analyzed in the N-telopeptides collagen chains.

In order to answer the question as to what extent potential sites for LOX-mediated cross-linking are present and conserved among metazoan lineages, we inspected the sequences of fibrillar collagens involved in the cross-linking reaction, namely the N- and C-telopeptides, and the corresponding C- and N-helical segments. C-propeptide sequences are the most conserved region of the fibrillar collagens, and the pattern of conservation of cross-linking sites can be analyzed by looking at the sequences downstream of the (GPP)_n_ repeats, the motif marking the C-terminus of the triple helix^23^. We searched for sequence similarities for the human α1 (I) or α1 (II) C-telopeptide cross-linking sites: QE**K**AH and RE**K**GP (K being the lysine providing the ε-amino group), respectively, in several metazoan clade A collagens as homology within this clade had been previously reported^8^. Multiple sequence alignment shows the presence of homologous sequences among the groups analyzed, with the pattern X**K**X′X″, where X is any residue, **K** the lysine involved in the reaction, X′ is glycine (mostly) or alanine (both small, non-polar aminoacids), and X″ is proline in most of cases, as shown in the weighted logo (Fig. 1A and B). N-propeptide sequences are the most variable within collagen families, including, in addition to the N-telopeptide, a cysteine-rich repeat, the von Willebrand factor-type C (VWC) module, a thrombospondin N-terminal -like domain (TSPN), or, as in some invertebrates α chains, a whey acidic protein (WAP) or von Willebrand factor A domain (VWA) modules, among others^24^. In most cases, the presence of a short triple helix marks the beginning of the N-telopeptide. Sequence comparison shows that a certain degree of homology was also observed around the cross-linking site (Fig. 2A and B). As for C-telopeptide sequences, a significant number of species display the pattern X**K**X′X″, with little variations in X′ and X″, such as in human, abalone and hydra. In addition to local sequence homology around the cross-linking telopeptide lysines, none of the collagens illustrated have any lysine residue between the C-terminal ends of the short N-terminal or main helices onto the cross-linking telopeptide lysines. Given a lysine occurrence of 7.2% in proteins, this has a probability of 1.2 × 10^−9^ (0.928^275^) of occurring randomly across all 19 C-terminal telopeptide sequences, and a probability of 1.9 × 10^−6^ (0.928^176^) for the 14 sequences between the (latterly removed) N-terminal helix and the cross-link, and is therefore a conserved feature. The regions between the C-terminal cross-link lysine onto the end of the molecule, and between the N-terminal cross-link lysine onto the main helix are also very lysine-poor, with no other lysine within five residues of the cross-linking one and the majority of sequences having no other lysine at all.

**Figure 3:**
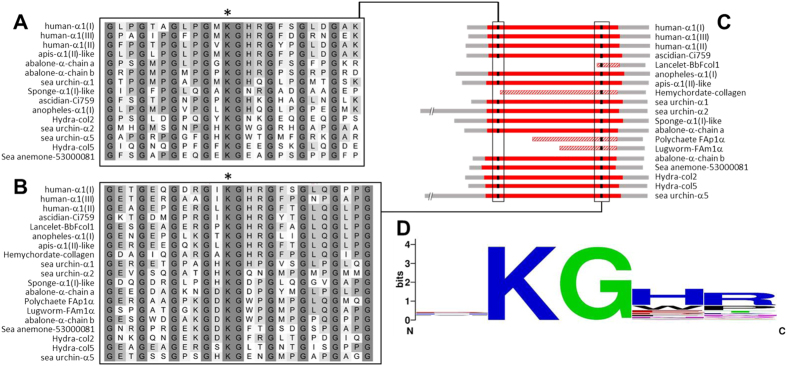
Sequence alignment of the helical cross-linking sites. Metazoan collagen chains used in Fig. 3 were aligned at the N- (**A**) and C- (**B**) ends of the central helical segment to search for homology at cross-linking sites. Panel C shows the location of the aligned segments, with the central helix marked in red. Available hemychordate, polychaete, lugworm and lancelet chains were incomplete at the N-terminal end. (**B**) Sequence logo of the position weight matrix of the putative cross-linking sites analyzed in the helical segments.

We also searched N- and C-terminal helix sequences for homology in the cross-linking sites. While the major triple helix is frequently interspersed with lysine residues, particularly in the third position of the collagen triplet, a recognizable and highly conserved pattern is observed at both N- and C-terminal helical cross-linking sites: Y**K**GY″Y″′ (Fig. 3A and B), where Y is any residue, **K** the lysine involved in the condensation reaction, the third position is invariably glycine as essential part of the collagen triplet repeats, and Y″ and Y″′ are histidine and arginine, respectively, in most of the cases.

## Discussion

**Figure 4:**
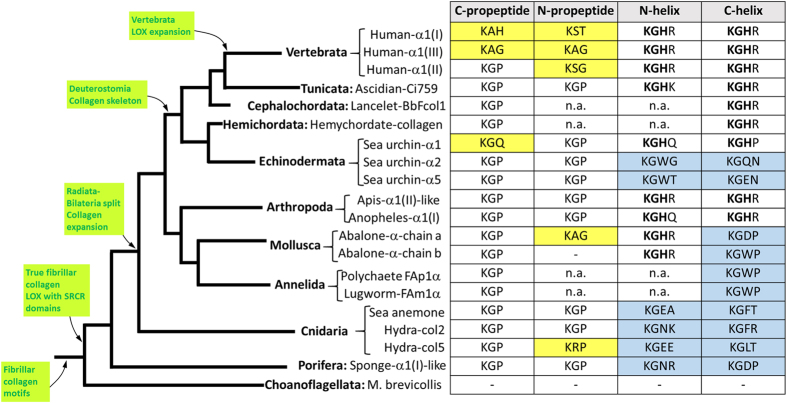
Evolution of collagen cross-linking sites across the tree of life. The figure combines the sequence information on the collagen cross-linking sites identified in the study and a cladogram representing a consensus view of the eukaryotic tree of life from the common ancestor of Metazoa and choanoflagellates. Yellow- and blue-colored table cells represent cross-linking sites in propeptides and helix, respectively, which are different from the consensus KGP (propeptide) and KGH (helix). Green boxes represent milestones in the evolution of fibrillar collagen and LOX enzymes. Blank cells indicate lack of homology, n.a.: sequences at those sites not available.

This study identified potential collagen cross-linking sites, which are conserved throughout the metazoan lineage. Several assumptions can be raised when these patterns of sequence conservation are analyzed across the tree of life (Fig. 4). First, these sequences were not found in the triple helix- or COLFI-containing polypeptides of the choanoflagellate *Monosiga brevicollis*. As this organism has been described not to express true fibrillar collagen, the acquisition of potential cross-linking sites is genuinely linked to the ability to produce this important matrix constituent^18^,^22^,^25^. This observation, together with the fact that LOX domains were shown to have a pre-metazoan origin, suggests that LOX enzymes might have been co-opted for collagen cross-linking in the lineage leading up to the Metazoa, presumably contributing to the formation of the ancestral fibrillar collagen. According to our study, N- and C-propeptide KGP sites first appeared in sponges, where fibrillar collagen has been identified to form the mesohyl, the central cavity that acts as an endoskeleton, supporting the tubular shape of sponges^26^. Interestingly, the acquisition of these cross-linking sites is coincident with the appearance of LOX proteins containing scavenger receptor cysteine-rich (SCRC) domains, suggesting these domains were key for the co-option of LOX enzymes to the remodeling of fibrillar collagens^22^. In fact, with the exceptions of some hydra, abalone and sea urchin chains, N- and C-propeptide KGP sites remain invariably until the appearance of vertebrate collagens, when this triplet experiences numerous changes (KAH, KAG, KST, KSG). From a LOX perspective, these variants were associated with new LOX protein architectures, without the SRCR domains and with propeptide and proline-rich regions, presumably contributing to the expansion of vertebrate collagens. Our study also shows different patterns of conservation in propeptide sites with respect to those in helical segments. This can be explained by the fact that helical lysines or hydroxylysines are not directly modified by LOX, but rather they are acceptor sites for the attack of telopeptide aldehydes, without participation of LOX enzymes. This circumstance likely determined distinct evolutionary events compared to those having occurred in the propeptides. With the exception of some sea urchin chains, our analysis shows a transition from sequences displaying only KG as conserved pattern, to those with KGH, with some organisms having both, one in N-helix and another in C-helix, such as the abalone chains. Considering that KG doublet is very frequently found in the helical segments, the acquisition of the specific KGH pattern might have contributed to fix the length of the helix and thereby its orientation within the supramolecular structure of the fiber^21^. Interestingly, this evolutionary transition predated the Radiata-Bilateria split, when a significant expansion of collagen chains has been proposed to occur^14^. Therefore, a model for the acquisition and evolution of cross-linking sites in fibrillar collagen is presented here that suggests, on one hand, that sequences becoming the target for LOX-catalyzed oxidation appeared soon in the metazoan lineage, and were invariably conserved until vertebrate LOX and collagen expansion. On the other one, helical cross-linking KGH sites might have fueled bilaterian evolution by strengthening and fixing the length and the structural orientation of collagen chains.

Collagen triple helix repeat-containing proteins have been also identified in bacteria and other non-metazoan forms of life, for which the formation of fibrils and higher order structures has not been described^27^,^28^,^29^,^30^. These collagen-like sequences, proposed to be horizontally transferred from metazoan, are always flanked by non-collagenous domains with structural motifs of surface or spore-associated proteins, which lack of a recognizable signature for the action of LOX enzymes, making highly improbable the formation of initiation products, and hence, the subsequent steps to the generation of covalent cross-links. Therefore, the identification of a highly conserved pattern of potential cross-linking sites in the C- and N-terminal domains is strongly associated to metazoan collagen chains, presumably having contributed to the formation and expansion of fibrillar collagens.

Solid biochemical data to support these assumptions is missing and there is only limited and very fragmented information about the existence of collagen cross-links in several organisms. For example, LOX-generated cross-links have been isolated from a sponge (*Haliclona oculata*), a sea urchin (*Strongylocentrotus droebachensis*), sea cucumbers (*Sclerodactyla briarius and Holothuria forskali*), and some cephalopods (*Loligo vulgaris* and *Sepia officinalis)* as well as from several annelids and molluscs^31^,^32^,^33^,^34^. Indirect evidences also suggest the cross-linking of collagen in other organisms. In the cnidarian *Hydra vulgaris*, which encodes six type I-like chains (Hcol1 to Hcol6), lathyric agents, well-known inhibitors of collagen cross-linking, have been shown to impair head regeneration, an evidence indicating a role for this collagen modification in *Hydra* morphogenesis^35^,^36^,^37^. Putative cross-linking sites were also identified in the marine polychaetous annelid *Alvinella pompejana*, for whose structural material a high thermal and chemical stability has been described, probably reflecting a high degree of cross-linking^38^. Nevertheless, it should be taken into account that other enzymes different that LOX, such as transglutaminases, have been also reported to cross-link collagens and other matrix proteins, and therefore, LOX-mediated remodeling might not be the exclusive route to a high stability^39^.

From a structural point of view, sequence requirements for LOX activity have been studied *in vitro* using synthetic oligopeptides^40^. By providing polyglycine peptide substrates to purified LOX in enzymatic activity assays, these authors investigated the influence of sequences found in the N- or C-telopeptides of mammalian α1(I) collagen chains. While the C-telopeptide sequence –QEK- was moderately favored when compared to the corresponding peptide with the lysine residue alone, the N-telopeptide sequence –DEK– was not oxidized at all. Although this study did not analyze the contribution of residues C-terminal to the reactive lysine, found to be highly conserved in our work, these observations reflect a higher degree of complexity in the recognition of collagen chains by LOX than that brought about by short peptides in solution. To this respect, LOX enzymes have been shown to be more active with collagen forming fibrils than with solubilized forms as a substrate, and more importantly, their binding capacity highly influenced by collagen-binding proteins such as small leucine-rich proteins (SRLP)^11^,^12^,^41^. Closely linked to this, LOX enzymes are also responsible for the cross-linking of tropoelastin monomers during the process of formation of elastic fibers, and sequence recognition within the tropoelastin chain very much differs from that observed in collagens^42^. Here too, the specificity of LOX on elastin substrates is complex and highly regulated by structural constraints and ancillary proteins, such as fibulins and fibrillins^43^,^44^,^45^. Interestingly, from an evolutionary perspective, it is much more evident that LOX were co-opted for a new function as elastin appeared much later in evolution compared to collagens and other matrix proteins^46^.

As a conclusion, this article has attempted to provide a general view of the process of collagen cross-linking by LOX enzymes, as well as to address the question about how this post-translational modification might have been incorporated into the collection of collagen biochemical reactions during evolution. Based on the identification of putative LOX-mediated cross-linking sites throughout the metazoan lineage, we propose that pre-existing LOX enzymes might have been co-opted for collagen cross-linking at the dawn of the Metazoa, thereby promoting the intermolecular association of ancestral collagen chains and the subsequent impact on their mechanical properties. Further refinement of N- and C- telopeptide cross-linking sites in vertebrates was associated with the appearance of new LOX genes likely contributing to collagen expansion, while helical sites might have evolved into the highly conserved KGH sequence to support the structural properties of the collagen chain, confirming the previous assumption that cross-linking has played a fundamental role in the evolution of the collagen fibril^21^. It should be nevertheless taken into account that this in silico analysis has only considered sequences in the clade A collagens, deliberately excluding those belonging to clade B, such as collagen XI (fibril-forming) or IX (fibril-associated), described also to be the subject of LOX-mediated cross-linking^8^. During the next years, further molecular and evolutionary genomic analyses will permit to get more insight about the contribution of this chemical modification to the evolution of the collagen fibrils.

## Methods

Collagen sequences used in the analysis were:

     Human α1 (I) [Genbank: AAB94054].

     Human α1 (III) [AGL34959].

     Human α1 (II) [NP_001835].

     Sea urchin-α1 collagen precursor *Strongylocentrotus purpuratus* [NP_999674].

     Ascidian-Ci759 fibrillar collagen α chain *Ciona intestinali*s genome: jgi-psf.org/ciona4/ciona4.home.html [ciona4|150759|ci0100150759].

     Sea urchin α2 collagen *Strongylocentrotus purpuratus* [NP_999675].

     Sea urchin α5 collagen *Paracentrotus lividus* [CAE53096].

     Anopheles-α1 (1) *Anopheles sinensis* [KFB49448].

     Apis-α1(II)-like *Apis mellifera* [XP_393523].

     Abalone-α-chain a *Haliotis discus* [BAA75669].

     Abalone α-chain b *Haliotis discus* [BAA75668].

     Lancelet-BbFcol1 fibrillar collagen *Branchiostoma belcheri*] [BAD97679].

     Polychaete FAp1a fibrillar collagen chain *Alvinella pompejana* [AAC35289].

     Sea anemone-53000081 collagen α-chain *Nematostella vectensis* genome: jgi.doe.gov/Nemve1/Nemve1.home.html [Nemve1|204742|fgenesh1_pg.scaffold_53000081].

     Hemichordate fibrillar collagen *Saccoglossus kowalevskii* [ABB83364].

     Hydra-col2 *Hydra vulgaris* [ABG80449].

     Hydra-col5 *Hydra vulgaris* [ABG80451].

     Sponge-α1(I)-like collagen *Amphimedon queenslandica* [XP_003388783].

     Lugworm-FAm1α fibrillar collagen *Arenicola marina* [AAC47545].

     *Monosiga brevicollis* [XP_001748906] and [XP_001749460] with triple helix, [XP_001744748], [XP_001747370] and [XP_001744747] with COLFI domains.

     Sequence alignments are available by the corresponding author upon request.

## Additional Information

**How to cite this article**: Rodriguez-Pascual, F. and Slatter, D. A. Collagen cross-linking: insights on the evolution of metazoan extracellular matrix. *Sci. Rep.* **6**, 37374; doi: 10.1038/srep37374 (2016).

**Publisher's note:** Springer Nature remains neutral with regard to jurisdictional claims in published maps and institutional affiliations.

## Supplementary Material

Supplementary Information
