# Supplementary material for: Collagen cross-linking: insights on the evolution of metazoan extracellular matrix
Source: Sci Rep. 2016 Nov 23;6:37374. doi: 10.1038/srep37374 (PMC5120351; doi:10.1038/srep37374)
Supplement: Supplementary Information [file srep37374-s1.pdf]

Revised version SREP-16-20944

Collagen cross-linking: insights on the evolution of metazoan extracellular matrix

Fernando Rodriguez-Pascual[1]* and David Anthony Slatter[2]

[1]Centro de Biología Molecular "Severo Ochoa" Consejo Superior de Investigaciones Científicas (C.S.I.C.) / Universidad Autónoma de Madrid (Madrid), Madrid, Spain.

[2]School of Medicine, University of Cardiff, Cardiff, United Kingdom.

Supplementary Information

Supplementary Figures 1 and 2.

Legends to Supplementary Figures

Supplementary Figure 1. Fibrillar collagen formation. Fibril-forming collagens (I-III, V, XI, XXIV, and XXVII) are synthesized as precursor forms with a central helical domain flanked by non-collagenous N- and C-propeptides. Chain recognition mediated by the C-terminus initiates the triple helix formation, which extends towards the N-terminal end in a zipper-like fashion. When the triple helix is completed, the so-called procollagen transits the Golgi network where it is packaged into secretory vesicles prior to export into the extracellular medium. After processing of the N- and C-propeptide regions by proteases of the ADAMTS and BMP-1/Tolloid families, respectively, collagen molecules self-assemble to form long fibrils with a characteristic axial periodic structure. Finally, the formation of covalent cross-links between lysine and hydroxylysine residues in the helix and telopeptides of adjacent molecules, a process initiated by members of the LOX family, is critical in providing the fibrils with strength and stability.

Supplementary Figure 2. Major cross-linking pathways of collagen. Once the N- and C-propeptide regions are cleaved off by the corresponding proteases, telopeptide lysine or hydroxylysine residues become accessible for the sequential reactions leading to the formation of cross-links. In type I collagen, the α1 chain C-telopeptide lysine/hydroxylysine (ct-Lys, ct-Hyl) and those in the α1 or α2 chain N-telopeptide (nt-Lys, nt-Hyl) are oxidatively deaminated by LOX enzymes, yielding the corresponding aldehydes t-Lys^{ald} and t-Hyl^{ald}, the initiation products for the cross-linking formation. Without the concourse of LOX in further reactions, these telopeptide aldehydes react with helical lysines or hydroxylysines to form immature cross-links, including, the Schiff-base intermolecular link dehydro-lysino-norleucine (deH-LNL) formed from either nt-Lys^{ald}/helical Lys 930 (933 if α2 chain), or from ct-Lys^{ald}/helical Lys 87. As these lysines are commonly hydroxylated, especially in stiffer connective tissues, cross-links may also be

dehydro-hydroxylysino-norleucine (deH-HLNL) from Lys^{ald} and Hyl, lysino-5-oxonorleucine (L5ONL) from Hyl^{ald} and Lys, or hydroxylysino-5-oxonorleucine (HL5ONL) from Hyl^{ald} and Hyl. An intramolecular aldol condensation product (AcP) can be also formed from two nt-Lys^{ald}. These Schiff bases and the AcP, even rearranged, can hydrolyse back to component amino acids. To form permanent cross-links, Schiff base cross-links react with each other and remaining Lys/Hyl over months/years to form trivalent pyrroles (adding Lys) and pyridinolines (adding Hyl). Alternatively, the UV-resistant trivalent histidino-hydroxylysinorleucine (HHL) forms from α2 His-92 and deH-HLNL in skin, whilst the AcP has been suggested to react with helical histidine and hydroxylysine to form the tetravalent dehydro-histidinohydroxy-merodesmosine (deH-HHMD), which is possibly a reduction artefact.

Supplementary Figure 1

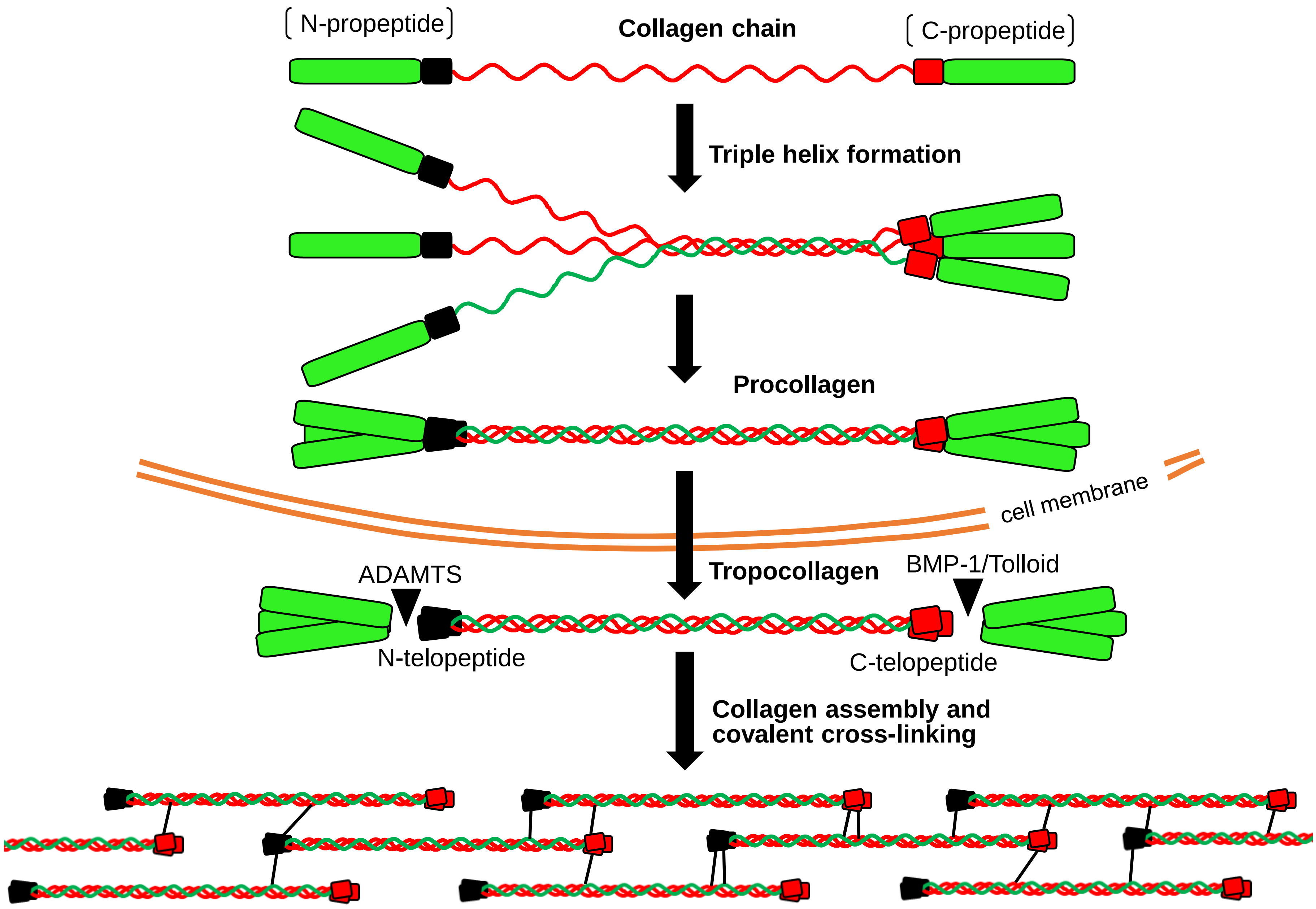

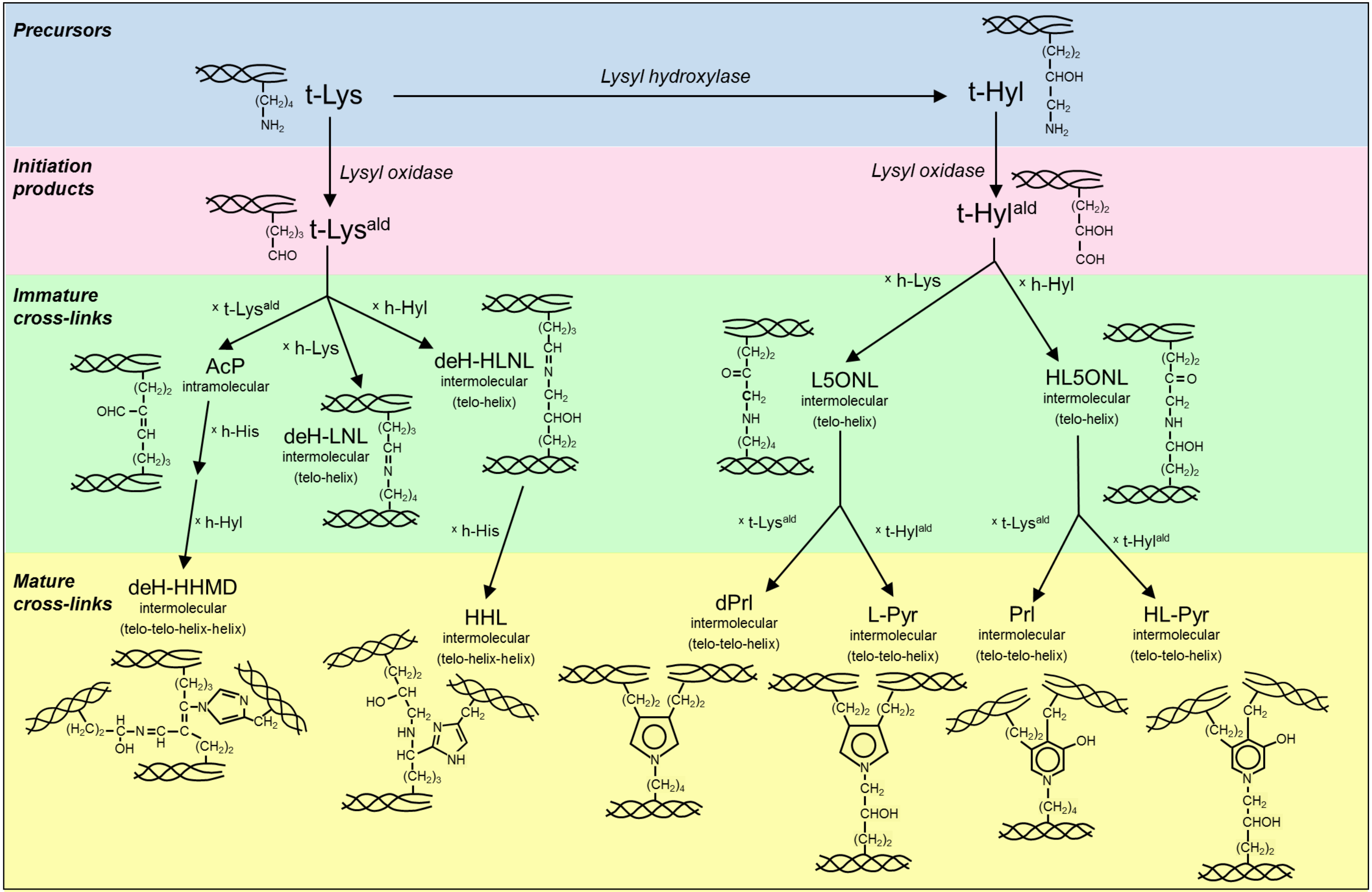

Supplementary Figure 2
